# Death of Professor Turner

**Published:** 1836-01-01

**Authors:** 


### Professor Turner.

It is with feelings of the deepest regret that we record the death of Mr. John William Turner, Professor of Surgery in the University of Edinburgh, after a short, but severe illness, brought on by exposure to wet and cold during the stormy night between Sunday 25th, and Monday 26th ult. Mr. Turner was called out of bed on that night, in the discharge of his duty as one of the Surgeons of the Royal Infirmary, and was detained upwards of two hours in wet clothes. He was unwell from that time, but did not confine himself, and continued to be occupied with his professional visits, and latterly with the duties of his Class of Surgery, till Friday, November 6th. From this period, his complaints, which at first assumed a dropsical tendency, became more complicated and aggravated, till Thursday, November 12th, when the relief he experienced from the remedies employed, encouraged his anxious family and medical attendants to indulge the hope that his valuable life might yet be spared; but this relief was of short duration, and after a few more days of severe illness, he died on the evening of Thursday, November 19th, deeply lamented both in his private and public capacity. He is succeeded by Sir Chas. Bell.

